# Shifting Gear in the Study of the Bilingual Advantage: Language Switching Examined as a Possible Moderator

**DOI:** 10.3390/bs9080086

**Published:** 2019-08-15

**Authors:** Evy Woumans, Shauni Van Herck, Esli Struys

**Affiliations:** 1 Department of Experimental Psychology, Ghent University, 9000 Ghent, Belgium; 2 Onderzoeksgroep Experimentele Oto-rino-laryngologie, KU Leuven, 3000 Leuven, Belgium; 3 Brussels Institute for Applied Linguistics, Vrije Universiteit Brussel, 1050 Brussels, Belgium

**Keywords:** bilingual advantage, executive control, language switching, shifting, inhibition, self-reports

## Abstract

The bilingual advantage is a heavily debated topic in research on bilingualism. The current study further investigated one specific aspect of bilingualism proposed to be a determining factor for the bilingual advantage, namely language switching behaviour. We investigated whether a bilingual advantage can be detected in the executive functions of inhibition and shifting by comparing monolingual and bilingual participants on a Simon task and a colour–shape switching task. Furthermore, we examined the relation between these executive functions and language switching proficiency, as measured by a semantic verbal fluency task. In addition, the current study set out to investigate the convergence of self-reported language switching estimates and actual language switching proficiency. Results revealed a bilingual advantage for shifting, but not for inhibition. However, this bilingual advantage for shifting was not related to language switching behaviour. Additionally, we were unable to identify a relation between objective and subjective measures of switching abilities. These findings seem to confirm the existence of a bilingual advantage, but also once again validate its elusiveness, as demonstrated by the absence of bilingual benefits on our measure of inhibition. It furthermore questions the validity of switching measures employed in previous studies.

## 1. Introduction

People tend to benefit from being bilingual in one way or another, but one beneficial consequence of bilingualism in particular has gained a lot of attention over the past two decades. This is the possible effect that controlling two or more languages might have on cognitive or executive control. This type of mental control is carried out by executive functions, which can be defined as “general-purpose control mechanisms that modulate the operation of various cognitive sub-processes and thereby regulate the dynamics of human cognition” [1] (p. 50). Miyake and colleagues proposed an influential subdivision of executive functioning into three main processes: inhibition, shifting and updating. Positive effects of bilingualism on measures quantifying executive control has been coined “the bilingual (cognitive) advantage”. The nature of the advantage is proposed to stem from a bilingual’s need to constantly and simultaneously govern two competing languages (e.g., by Bialystok and colleagues [2])

Bilingual advantages have been observed in all three subdomains of executive functioning. For instance, Carlson and Meltzoff [3] administered nine executive function measures to children attending kindergarten school and found that bilingual children had significantly higher scores than monolingual children on tasks that required inhibition of attention to prepotent or distracting responses. This bilingual advantage in inhibition can be accounted for by the Inhibitory Control (IC) model, proposed by Green [4]. This model proposes that the inhibitory mechanisms involved in suppressing one language when activating the other are suggested to be domain-general, and might therefore exert an influence on tasks tapping into nonverbal and general inhibitory control. Outperformance for bilinguals over monolinguals has also been disclosed for measures of updating, sometimes also referred to as monitoring. It was Costa and colleagues [5] who put forth that bilinguals are better at resolving inhibitory tasks that require conflict resolution due to their improved monitoring system. Indeed, their study showed that bilinguals not only exceeded monolinguals on experimental trials involving conflict (e.g., the incongruent trials in response inhibition tasks), but also on trials requiring no conflict resolution at all (i.e., congruent trials). They attributed this better performance to bilinguals’ need for enhanced monitoring mechanisms, which arise from the constant monitoring of languages that takes place when a bilingual engages in conversation. Lastly, bilingual benefits have been reported for measures of shifting. For instance, Prior and MacWhinney [6] compared monolingual and bilingual college students on a task-switching paradigm and reported significantly reduced switching costs for bilinguals. This switching or shifting advantage is compatible with the hypothesis that cognitive benefits in bilinguals are more likely to appear on tasks similar to bilingual language use, such as task-switching paradigms [7].

Even though the studies reported thus far all seem to point in the direction of a bilingual advantage, more recently, some authors have started to challenge these findings [8,9,10,11]. One particular study that caused a great stir in the then already ongoing debate was conducted by Paap and Greenberg [12]. This seminal paper compared monolinguals and bilinguals on 15 indicators of executive processing and reported no bilingual benefits whatsoever. Moreover, results from one task even disclosed a bilingual disadvantage. A later study by Paap and colleagues [11] was a bit more nuanced and suggested that the bilingual advantage may be restricted to specific and undetermined circumstances. Additionally, de Bruin and colleagues [8] provided evidence for a publication bias favouring research demonstrating better performance of bilinguals over monolinguals. Nevertheless, although this meta-analysis claimed that there was no clear evidence for a bilingual advantage, its results did reveal that studies using Simon tasks had a very high probability of detecting large effects of bilingualism (i.e., an average of 0.87 to detect d of 1.08, and 0.99 to detect d of 2.99).

As a result of these discrepant findings, some authors have argued that the yes/no debate on the existence of the bilingual advantage has not been very productive. Instead, we should move toward investigating its possible moderating factors [13]. It has been argued that one such factor may be language switching behaviour [14,15,16,17]. To illustrate, Prior and Gollan [14] compared a group of Spanish–English bilinguals who often switched between their two languages with a group of Mandarin–English bilinguals who switched between their two languages less often. Their results demonstrated that the bilingual population that often switched between their languages outperformed a group of monolinguals as well as the nonfrequent switchers on a nonverbal switching task. Verreyt and colleagues [16] built upon these findings by comparing three groups of Dutch–French bilinguals (unbalanced bilinguals, balanced nonswitching bilinguals and balanced switching bilinguals) on two other executive tasks measuring inhibitory control. Results indicated that the balanced switching bilinguals outperformed both the balanced nonswitching bilinguals and the unbalanced bilinguals, with the balanced switching bilinguals demonstrating smaller congruency effects than the balanced nonswitching bilinguals. Additionally, Verreyt and colleagues [16] demonstrated negative correlations between language switching frequency and conflict resolution skills (r = −0.388 and r = −0.258, respectively). Hence, whereas Prior and Gollan [14] established that some sort of transfer takes place from verbal switching practice to the more general nonverbal executive function of switching, the study by Verreyt and colleagues [16] suggested even further transfer to include the executive functions of interference resolution or inhibition. 

In addition to self-reported and thus subjective measures, recent research has started using objective measures to assess the effects of language switching proficiency on cognitive processing. To this end, Woumans and colleagues [17] employed a semantic verbal fluency task with two conditions; a single-language and a dual-language condition. In the single-language condition, participants had to produce words of certain semantic categories (e.g., animals) in one of their languages for 60 s. In the dual-language condition, participants were asked to continuously switch between their two languages. The researchers employed measures of inhibition and attention to relate to the ability of language switching. The results of their study pointed toward an advantage of bilingual groups (unbalanced, balanced and interpreters) over the monolingual group, and a correlation between switching proficiency as measured by the fluency task and conflict resolution in the group of balanced bilinguals (r = 0.530). This substantiated the claim made by previous research, such as that by Soveri and colleagues [15], suggesting lifelong experience with language switching as a determining factor for the bilingual advantage.

The Present Study. As the abovementioned research revealed a possible moderating effect of language switching on the bilingual advantage phenomenon, the aim of the present study was to investigate the effect of this linguistic variable on two measures of executive function: inhibition and shifting. We therefore compared monolingual and bilingual participants on the Simon task (originally described by Simon and Rudell [18], for a review see Lu and Proctor [19]), tapping into inhibition; and the colour-shape switching task (originally described by Rubin and Meiran [20]), tapping into shifting. Looking at the previous findings, it was our hypothesis that language switching particularly contributes to a possible bilingual advantage over monolinguals, and we therefore predicted a correlation between language switching proficiency in bilinguals and our measures of executive functioning. An additional asset of the current study is that next to the implementation of an objective measure of switching proficiency (as measured by an adapted version of the semantic verbal fluency task), we also obtained a measure of self-reported switching practice (i.e., frequency of language switching within conversations). We expected both switching proficiency and switching practice to correlate positively with better performance on the Simon task and the colour-shape switching task.

An additional aim of this study was to examine how closely these two measures of language switching (i.e., proficiency and practice) correlate with one another. Yim and Bialystok [21] already demonstrated a relationship between the amount of language switching taking place in a single conversation and performance on another adapted version of the verbal fluency task. If people accurately report how many times they approximately switch between their languages in conversation, there should also be a correlation between performance on the semantic verbal fluency task and the score for language switching practice (i.e., frequency) on the language questionnaire.

## 2. Materials and Methods

Participants. Thirty-four undergraduate psychology students at Ghent University (Belgium) participated in this study for course credit. This group consisted of 16 monolinguals and 18 bilinguals, and all spoke Dutch as their native language. This grouping was based on the self-reported scores on the language questionnaire. Participants were considered monolingual if their composite score on the language questionnaire for the proficiency in L2 (comprehending, speaking, reading and writing) was weak to intermediate (i.e., a score of 3 or less, but preferably a score of 2 or less). The bilinguals’ L2 was either English, French, Spanish, Polish or Turkish. Proficiency in L1 and L2 was also objectively measured by means of the single-language blocks in a semantic verbal fluency task. Monolinguals and bilinguals were required to be equally proficient in L1 (Dutch), having learnt the language form birth, using it both at home and in a school context. Detailed demographic data of the monolingual and bilingual groups are presented in Table 1.

**Language questionnaire.** All participants completed a language questionnaire, loosely based on the instrument designed by Verreyt and colleagues [16], but adapted for the purposes of the present study. This questionnaire examined language proficiency and switching behaviour, but also included a measure of socioeconomic status (SES). Participants were asked to indicate which language they speak as L1 (this is Dutch for all participants) and indicate all other languages of which they have any knowledge. Furthermore, they had to specify in which contexts they used these languages and the age at which they acquired them. Proficiency was indicated for comprehending, speaking, reading and writing on a 5 point Likert-scale, ranging from 1 (=no proficiency) to 5 (=perfect/native speaker level). An overall proficiency score was calculated by taking the average of the proficiency scores for all four language skills. Further questions tapped into bilinguals’ language switching behaviour, including inquiries into switching context and frequency of language switching within conversation (1 = never, 5 = constantly). Participants were also asked to indicate their parents’ educational level, which was taken as a proxy for SES. Possibilities were primary education, lower secondary education, higher secondary education and higher education.

**Semantic verbal fluency task.** To the end of assessing verbal fluency in one or two languages, we implemented a semantic fluency task adapted from the one employed by Woumans and colleagues [17]. This task was taken as an objective (but not necessarily naturalistic, see below) measure of both language and language switching proficiency. Specifically, participants were given 60 s to name words belonging to a certain semantic category (animals, vegetables or professions). The task consisted of a single-language condition and a dual-language condition. In the single-language condition, participants were restricted to producing words in one specific language (either L1 or L2). Performance in the single-language blocks was taken as a measure of fluency in these respective languages. In the dual-language condition, participants were required to continuously alternate between their L1 and L2 when naming the words within the given category. Monolinguals performed all three categories in their L1 because even though they had been exposed to (an)other language(s) than their L1, their proficiency in that (or those) language(s) based on the self-reported scores was considered too low to perform the task in that (or those) language(s). Bilinguals, on the other hand, completed the task for their respective language pairs. They performed one category in L1, one category in L2 and one category switching between the two. Categories as well as the language order in which the categories were performed were counterbalanced across participants. The dual-language condition was always completed last. For the bilinguals, a language switch cost was calculated on the basis of the number of words produced in the L1 single-language condition and the number of L1 words produced in the dual-language condition. This was then taken as a measure of language switching proficiency. A small language switch cost indicated a fluent switcher, a large language switch cost implied a nonfluent switcher (see also [17]).

**Simon task.** To assess inhibition, a Simon task similar to that in the study of Woumans and colleagues [17] was used. Here, coloured dots appeared on either the left or the right side of the screen. Participants had to respond to the colour of the dot. They were instructed to press the left arrow on the keyboard when a red dot appeared and the right arrow when a green dot appeared. Response mapping was counterbalanced across participants. Trials were congruent if position and colour of the dot elicited the same response and incongruent if the position and colour elicited different responses. 

Trials started with a fixation cross, which appeared on screen for 500 ms, followed by a blank screen. Afterwards, a red or green dot appeared on either the left or the right side. The coloured dot stayed on screen until the participant’s response or for a maximum time of 900 ms. The screen turned blank for 500 ms before the next fixation cross appeared and the next trial started. The proportion of congruent/incongruent trials in this experiment was 50/50%, in line with previous research showing that the bilingual advantage is most prominently seen in this high-monitoring context, with many and unpredictable switches between congruent and incongruent trials, compared with low-monitoring contexts with an uneven distribution of congruent and incongruent trials and a lower number of switches between these two trial types ([5], but see also [22]). The experiment included 10 practice trials and two blocks of 100 experimental trials each. Stimuli were presented via PsychoPy v1.85.2 software in Python [23,24] on a laptop with a 15.6 inch screen.

**Colour**–**shape switching task.** To the end of assessing shifting, a colour–shape switching task was employed, similar to the one used by Prior and MacWhinney [6]. In this task, the targets were circles or triangles, which were either blue or yellow. During shape evaluation, participants had to decide whether the target was a triangle or a circle by pressing the designated keys on a keyboard with their index and middle finger of one hand. Similarly, when judging colour, they responded with the index and middle finger of the other hand. Which task was assigned to which hand was counterbalanced across participants. Independently of which hand they had to use for the given task, the index finger was always designated to the colour “blue” and the shape “circle”, whereas the middle finger to the colour “yellow” and the shape “triangle”. In single-task blocks, all trials were of the same type (either shape or colour). In mixed-task blocks, trials could be of both types and a task cue indicated which task the participants had to perform on the subsequent trial. 

A trial started with a fixation cross for 350 ms. After the fixation cross, the screen turned blank for 150 ms. Following the blank screen, there was a task cue on screen for 250 ms. In case of the colour task, the cue was the Dutch word for colour (“kleur”) displayed on screen. In case of the shape task, it was the Dutch word for shape (“vorm”). After 250 ms of task cue presentation, a target appeared on screen, while the task cue also remained. The target and cue stayed on screen until the participant responded, or for a maximum time of 4000 ms. The screen turned blank for 850 ms before the next fixation cross appeared and the next trial started. The participants first performed two single-task blocks (one for colour and one for shape), consisting of eight practice trials and 40 experimental trials. Subsequently they performed the mixed-task blocks. There was one block of 16 practice trials, and three blocks of 48 experimental trials. Of these trials, the proportion of switch/nonswitch trials was 50/50%. Following the mixed-task blocks, participants performed the two single-task blocks again, but in the opposite order. The order of the single-task blocks was counterbalanced across participants. Stimuli were presented via PsychoPy v1.85.2 software for Python [23,24] on a laptop with a 15.6 inch screen.

**Procedure**. When registering for the study, participants first completed the questions in the language questionnaire that were specifically aimed at assessing L2 proficiency. Upon arrival at the experiment, participants completed the informed consent, and subsequently performed the Simon task and the colour–shape switching task. Following this, they filled in the rest of the language questionnaire, and the semantic verbal fluency task was administered. The order of the Simon task and the colour–shape switching task was counterbalanced across participants. Task instructions were provided in Dutch.

## 3. Results

### 3.1. Background Data

Table 1 reports all analyses performed on the demographic variables, including language background. No differences were found between language groups (monolinguals vs. bilinguals) with regard to gender ratio, age, SES, L1 proficiency and age of L1 acquisition. There were, however, significant differences for frequency of L1 use, L2 proficiency, age of L2 acquisition, frequency of L2 use and language switching frequency. Bilinguals employed their L1 less frequently and their L2 more frequently. They also acquired their L2 at an earlier age than monolinguals and reported higher proficiency. In addition, they more often switched between their languages within conversation.

### 3.2. Semantic Verbal Fluency Task

All data from the semantic verbal fluency task are reported in Table 1. There was no difference between the monolingual and bilingual group with respect to the number of words produced in the L1 condition. 

### 3.3. Executive Control Tasks

Data from the Simon task and colour–shape switching task are presented in Table 2. For the Simon task, three participants (two monolingual, one bilingual) were excluded from analyses because they scored below chance level (mean accuracy below 50%). For the colour–shape switching task, three participants (one monolingual, two bilingual) were also excluded because they scored below chance level (mean accuracy below 25%). Reaction times (RTs) were trimmed by excluding those for incorrect trials and those deviating more than 2.5 SD from the participant’s individual mean on that task. This resulted in a removal of 8% of all trials in the Simon task and 16% of all trials in the colour–shape switching task.

### 3.4. Simon Task

The Simon effect is defined as the difference in performance between congruent and incongruent trials. The Simon effect was analysed for RTs and accuracy using two-way repeated measures ANOVA, with Congruency as a within-subjects factor (congruent, incongruent) and Group as a between-subjects factor (monolingual, bilingual). RT analysis yielded a significant effect of Congruency [*F*(1, 29) = 51.15, *p* < 0.001], indicating a Simon effect with faster RTs on congruent trials. No effect of Group was present [*F*(1, 29) = 1.23, *p* = 0.275], neither was there a Group*Congruency interaction [*F*(1, 29) = 1.29, *p* = 0.268]. Accuracy analysis also revealed a main effect of Congruency [*F*(1, 29) = 33.99, *p* < 0.001], reflecting a Simon effect with higher accuracy on congruent trials. The effect of Group and the Group*Congruency interaction failed to reach significance [*F*(1, 29) = 0.01, *p* = 0.905; *F*(1,29) = 1.26, *p* = 0.258].

### 3.5. Color–Shape Switching Task

#### 3.5.1. Switching Cost

The switching cost is defined as the difference in performance between switch and nonswitch trials in the mixed-task blocks. Switching costs were analysed for RTs and accuracy using two-way repeated measures ANOVA, with Trial Type as a within-subjects factor (switch, nonswitch) and Group as a between-subjects factor (monolingual, bilingual). In the RT analysis, a significant main effect of Trial Type was detected [*F*(1, 29) = 116.07, *p* < 0.001], revealing a switching cost with faster RTs on nonswitch trials. The effect of Group was not significant [*F*(1, 29) = 0.93, *p* = 0.343]. However, RT analysis did disclose a significant Group*Trial Type interaction [*F*(1, 29) = 6.17, *p* < 0.05], which implied a smaller switching cost in RTs in the bilingual group compared with the monolingual group. Accuracy analysis only exposed a significant main effect of Trial Type [*F*(1, 29) = 11.73, *p* < 0.01), reflecting a switching cost with a higher accuracy on nonswitch trials. The effect of Group and the Group*Trial Type interaction did not reach significance [*F*(1, 29) = 0.66, *p* = 0.413; *F*(1, 29) = 0.02, *p* = 0.969].

#### 3.5.2. Mixing Cost

The mixing cost is defined as the difference in performance between trials in the single-task blocks and nonswitch trials in the mixed-task blocks. Mixing costs for RTs and accuracy were analysed using two-way repeated measures ANOVA, with Trial Type as a within-subjects factor (single task trials, nonswitch trials) and Group as a between-subjects factor (monolingual, bilingual). RT analysis yielded a significant main effect of Trial Type [*F*(1, 29) = 26.43, *p* < 0.001], indicating a mixing cost with faster RTs on single task trials. The effect of Group and the Group*Trial Type interaction did not reach significance [*F*(1, 29) = 0.54, *p* = 0.468; *F*(1, 29) = 0.09, *p* = 0.768]. Accuracy analysis yielded no significant effects [Trial Type: *F*(1, 29) = 1.36, *p* = 0.278; Group: *F*(1, 29) = 0.99, *p* = 0.326; Group*Trial Type: *F*(1, 29) = 1.05, *p* = 0.300].

### 3.6. Language Switching Proficiency vs. Executive Control

#### 3.6.1. Simon Task

Correlation analysis yielded no relation between language switch cost, as a measure of language switching proficiency, and the Simon effect in RTs and accuracy (Simon RT: r = 0.38, *p* = 0.159; Simon accuracy: r = 0.09, *p* = 0.749). Accordingly, there was no relation between Simon effects in RTs and accuracy and L2 proficiency or SES.

#### 3.6.2. Colour–Shape Switching Task

No relations between language switch cost, as a measure of language switching proficiency, and switching and mixing costs in RTs and accuracy were detected (switch RT: r = 0.39, *p* = 0.146; switch accuracy: r = −0.23, *p* = 0.406; mix RT: r = 0.24, *p* = 0.395; mix accuracy: r = −0.03, *p* = 0.918). Neither was there a link between any of these executive control measures and SES or L2 proficiency.

### 3.7. Language Switching Proficiency vs. Language Switching Frequency

Among bilinguals, correlation analysis revealed no relation between language switch cost, calculated from the semantic verbal fluency task, and language switching frequency, as measured by the language questionnaire (r = −0.06, *p* = 0.827).

## 4. Discussion

Recently, researchers started to challenge the bilingual advantage, arguing that it either does not exist, or is restricted to very specific circumstances [8,11,12]. In response to this, some studies argued that given aspects of bilingual language use, rather than bilingualism itself, might be at the basis of the bilingual advantage. One such aspect may be language switching [14,16,17]. The aim of the current study was therefore threefold. Firstly, we aimed to examine whether a bilingual advantage can be detected by comparing monolinguals and bilinguals on a Simon task and a colour–shape switching task. Secondly, we wanted to investigate whether language switching behaviour is related to performance of bilinguals on these executive function tasks. Thirdly, we wanted to verify the relationship between objective language switching proficiency, as measured by the semantic verbal fluency task, and subjective language switching frequency, as measured by a language questionnaire.

Our results revealed a smaller switching cost in RTs in bilinguals relative to monolinguals in the colour–shape switching task. With respect to the switching cost in accuracy, the mixing costs and the Simon effects, we established no differences between the monolingual and bilingual group. In addition, within the bilingual group, language switching proficiency was not related to executive functioning. Furthermore, we failed to detect a relation between participants’ self-reported (and consequently more subjective) language switching frequency and their objective language switching proficiency.

The present study particularly challenges the existence of a bilingual advantage specifically for inhibitory control and is not the first in doing so [12,25]. Even though the probability of detecting an effect of bilingualism in a Simon task is high [26], our results do not converge with the findings of these previous studies. Nevertheless, we must acknowledge the fact that the monolinguals in the current study were not genuine monolinguals when defined as people with knowledge of only one language, because individuals who meet this strict criterion of monolingualism would be nearly impossible to recruit in a multilingual country such as Belgium. It is even possible that the difficulty of recruiting genuine monolinguals in modern-day cosmopolitan societies is one of the reasons behind the current replication crisis in research on the bilingual advantage in cognitive control [10]. Although we aimed for a sufficiently large difference in L2 proficiency between the group of monolinguals and the group of bilinguals, this may have influenced our findings. We therefore recommend future studies to also include a group of monolinguals with no or hardly any exposure to other languages, even though we admit that these individuals may be hard to find in most contexts and definitely in any country where English is not the majority language. Additionally, the number of participants per language group was limited, reducing the power of the statistical analysis and thus leading to some caution in the interpretation of the present results. Even so, this study did reveal a reduced switching cost for bilinguals compared with monolinguals on RTs in the colour–shape switching task. This finding is in line with previous studies suggesting bilingual benefits for shifting [6,14]; which supports the hypothesis posed by Bialystok and colleagues [7] that the bilingual advantage most likely appears on tasks that bear most similarity to bilingual language use, such as task switching paradigms. However, it should be recognised that a recent meta-analysis by Lehtonen and colleagues [10] did not find a reliable shifting advantage in bilinguals. Following up on previous research by Verreyt and colleagues [16], it could be interesting for future studies with a similar design to consider two further distinctions between balanced and unbalanced bilinguals, and between frequent and nonfrequent language switchers. It can be expected that these two variables of balanced proficiency and switching frequency may modulate the absence or presence of a bilingual advantage and its effect size. Moreover, in order to obtain more consistent outcomes, future studies are suggested to administer a variety of cognitive control tasks, such as the flanker task [5] or the AX-CPT (AX Continuous Performance Task) [27]. 

Previous studies also proposed language switching behaviour as an important factor driving the bilingual advantage [14,16,17]. Rather surprisingly, and especially in light of the bilingual advantage in shifting present in our results, the current study did not reveal a relation between language switching proficiency and executive functioning in bilinguals. Still, the surprising outcome of the present study may partially be explained by its relatively low sample size and lack of variation among participants on the language switching variable. Compared with the L2 proficiency variable, which was also measured on a 5 point Likert-scale, the range of responses and the standard deviation were much lower for the switching frequency variable. Somewhat similar to our results, Yim and Bialystok [21] were unable to identify a relation between language switching in conversation and nonverbal task switching. Considering that their results indicated a negative correlation between language switching in conversation and switch cost in the semantic verbal fluency task, we could expect that in their bilingual population, there would be no relation between nonverbal task switching and switch cost in the semantic verbal fluency task (and hence language switching proficiency). As Woumans and colleagues [17] did detect a negative correlation between switching proficiency and the Simon effect, the authors explained the discrepancy between their results and those of Yim and Bialystok [21] by stating that this relation may only be present in balanced bilinguals. Yim and Bialystok [21] analysed unbalanced and balanced bilinguals together; however, balanced bilinguals may be the only group sufficiently trained in language switching for it to have an effect on cognitive functioning. Since the bilingual group in the present study also contained both balanced and unbalanced bilinguals, this may explain the lack of a relation in our study.

A last and rather surprising finding of the current study is that we observed no relationship between language switching frequency, as reported by the participants in the language questionnaire, and their language switching performance on the verbal fluency task. In the past, multiple studies have relied on self-reported language switching frequency as a measure of language switching in bilinguals [14,16]. However, it appears that these self-reported measures do not always converge with objective measures such as the semantic verbal fluency task. Even though the relation between self-reported measures and some other objective measures, such as picture naming tasks, has previously been confirmed [28,29], caution is warranted, and the relation between self-reported measures and objective measures should be more extensively studied in the future. One reason for the elusiveness of this relationship between objective and subjective measures of language switching may be that these objective measures do not necessarily reflect naturalistic code-switching in which bilinguals often engage. For instance, in the task used in the present study, bilinguals were expected to continuously switch back and forth between naming items in a specified semantic category, but this language behaviour is uncommon in real-life bilingual conversations, where code switching is not cued and occurs rather naturally. We therefore recommend future studies to use objective measures of language switching that align more with naturalistic language use in bilingual populations. Especially important in light of the more recent approach to the bilingual advantage in which the effects of language switching are examined, it is also recommended to broaden this research area to the validity of subjective measures of language switching.

## 5. Conclusions

Notwithstanding its formerly raised limitations, the current study has some important implications. Indeed, our research revealed a bilingual advantage in RT switching costs, indicating that bilingualism particularly influences the executive function shifting. The lack of a relation between the executive functions of the bilinguals and language switching proficiency showed that the bilingual advantage we detected is not necessarily related to the language switching proficiency of the bilinguals. This study also emphasizes the importance of objective measures of language abilities, by demonstrating that language switching proficiency and language switching frequency are, at least in the current sample, not related.

## Figures and Tables

**Table 1:** Demographic data of the monolingual and bilingual groups, reported as means, with standard deviations between parentheses.

	Monolingual	Bilingual	Test	*p*
N	16	18	N/A	N/A
Male/female ratio	1/15	3/15	Chi^2^ = 0.885	0.347
Age	18.56 (0.63)	19.82 (4.81)	*t* = −1.04	0.307
SES	2.63 (0.67)	2.67 (0.66)	*t* = −0.18	0.857
L1 proficiency	4.92 (0.22)	4.88 (0.20)	*t* = 0.66	0.515
L1 Age of acquisition	0.00 (0.00)	0.00 (0.00)	No difference	
L1 Frequency of use (%)	87.80 (9.66)	77.27 (10.56)	*t* = 2.53	0.019*
L2 proficiency	2.59 (0.36)	4.65 (0.38)	*t* = −15.98	<0.001*
L2 Age of acquisition	12.72 (1.34)	4.72 (5.20)	*t* = 6.30	<0.001*
L2 Frequency of use (%)	12.20 (9.66)	22.73 (10.56)	*t* = −2.53	.019*
Switching frequency	2.49 (0.28)	3.41 (0.51)	*t* = −6.37	<0.001*
L1 Verbal Fluency	19.06 (3.34)	19.5 (6.06)	*t* = −0.26	0.800
L2 Verbal Fluency	N/A	15.00 (4.00)	N/A	N/A
Switching cost	N/A	13.50 (5.71)	N/A	N/A

*Note.* Switching frequency was indicated on a scale from 0 (= never) to 5 (= constantly). L1 Verbal Fluency was the mean number of words produced in the L1 blocks in the semantic verbal fluency task, whereas L2 verbal fluency was the mean number of words in the L2 blocks. Switching cost constituted the mean difference between the number of words in the L1 block and the number of L1 words in the dual-language block. Significant differences are indicated with an asterisk. SES: socioeconomic status.

**Table 2:** Mean reaction times (RTs, ms) and accuracy (% correct) for the Simon task and colour–shape switching task, with standard deviations in parentheses.

	Simon			Colour–Shape Switching	
	Monolingual	Bilingual		Monolingual	Bilingual
RT			RT		
Congruent	436 (45)	420 (61)	Single	597 (139)	553 (112)
Incongruent	479 (49)	451 (58)	Switch	907 (185)	801 (254)
Congruency effect	43 (19)	31 (35)	Nonswitch	705 (132)	674 (213)
			Switching cost	202 (101)	126 (66)
			Mixing cost	108 (120)	121 (127)
% correct			% correct		
Congruent	97 (2)	96 (3)	Single	83 (20)	91 (18)
Incongruent	92 (4)	93 (4)	Switch	82 (16)	87 (17)
Congruency effect	4 (4)	3 (3)	Nonswitch	86 (15)	91 (18)
			Switching cost	4 (7)	4 (5)
			Mixing cost	– 3 (9)	0 (4)
